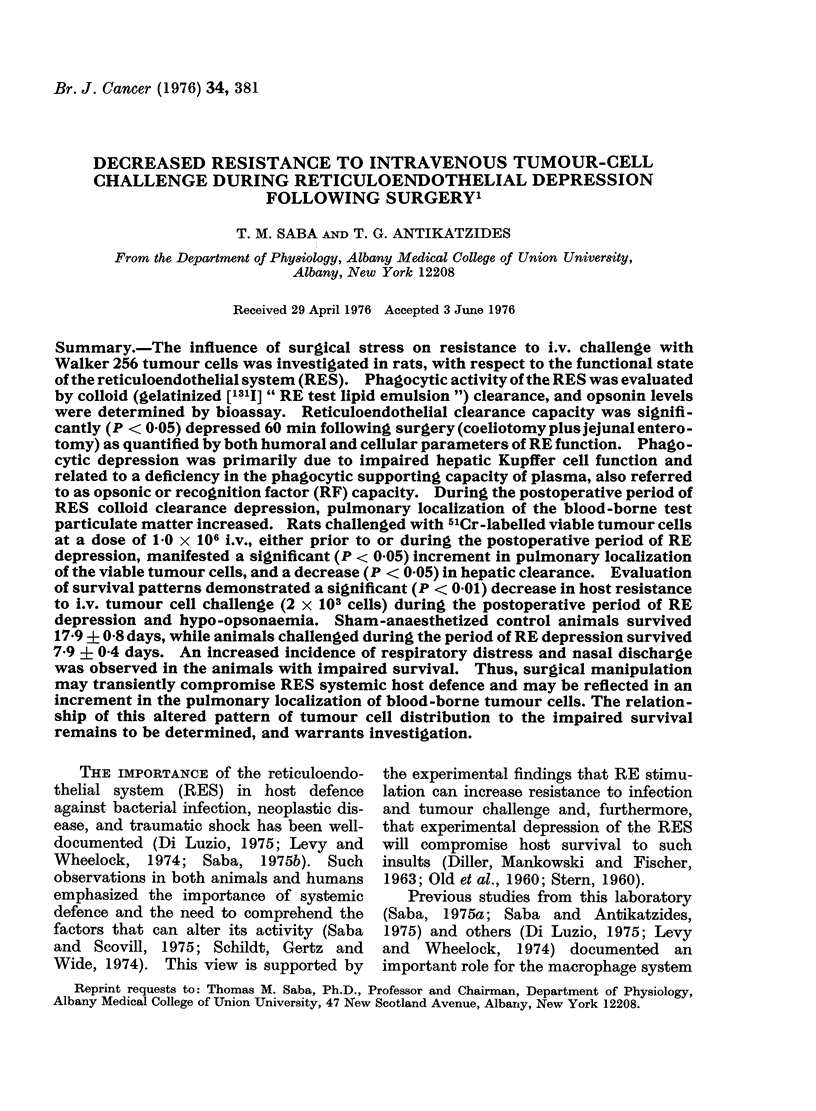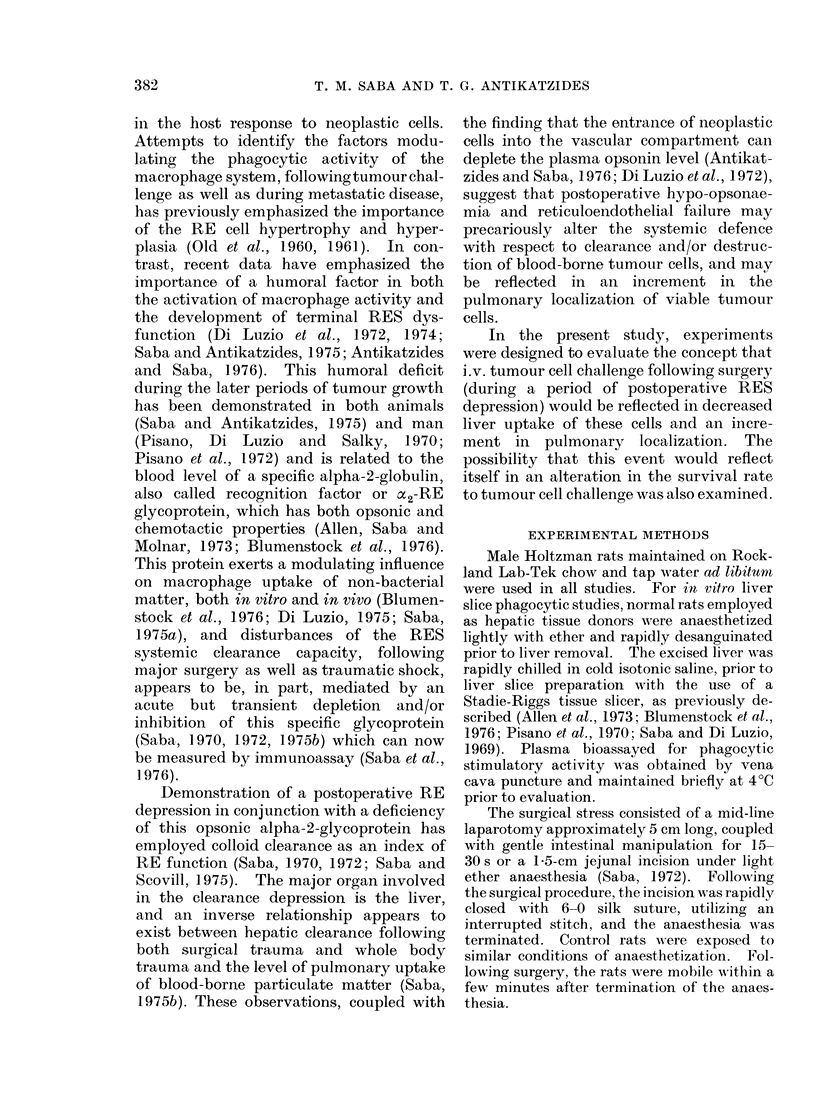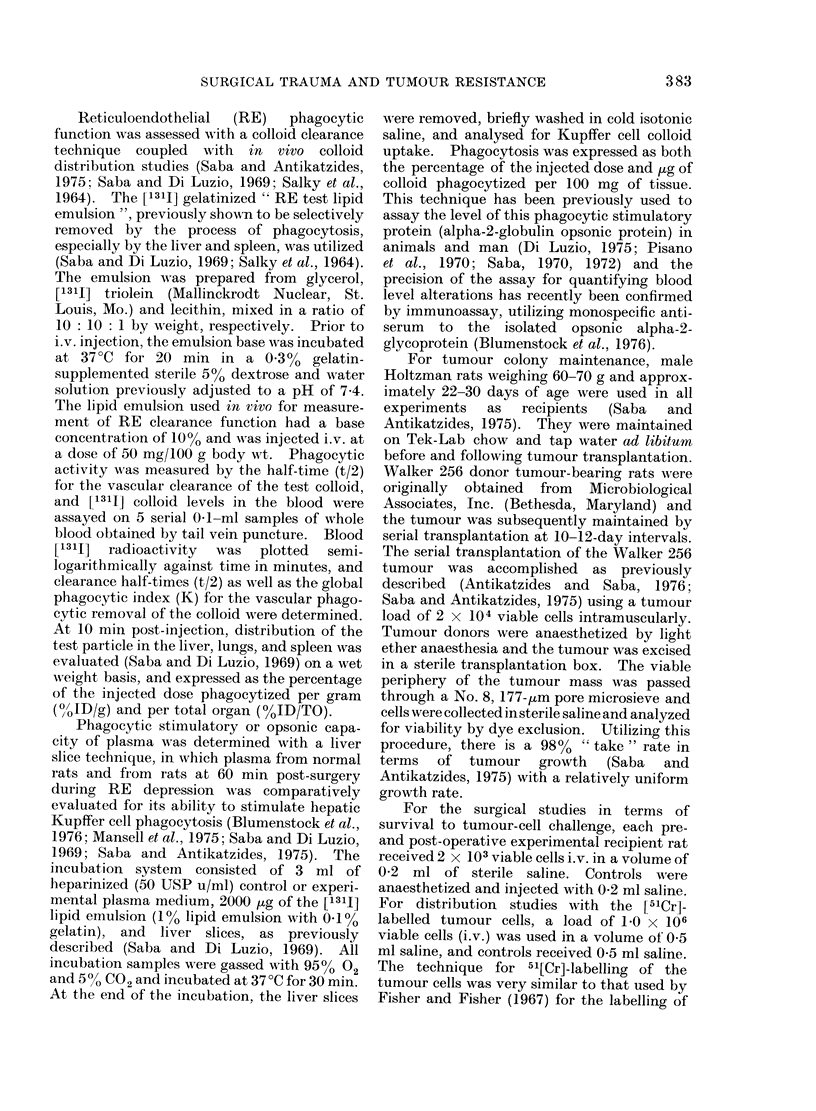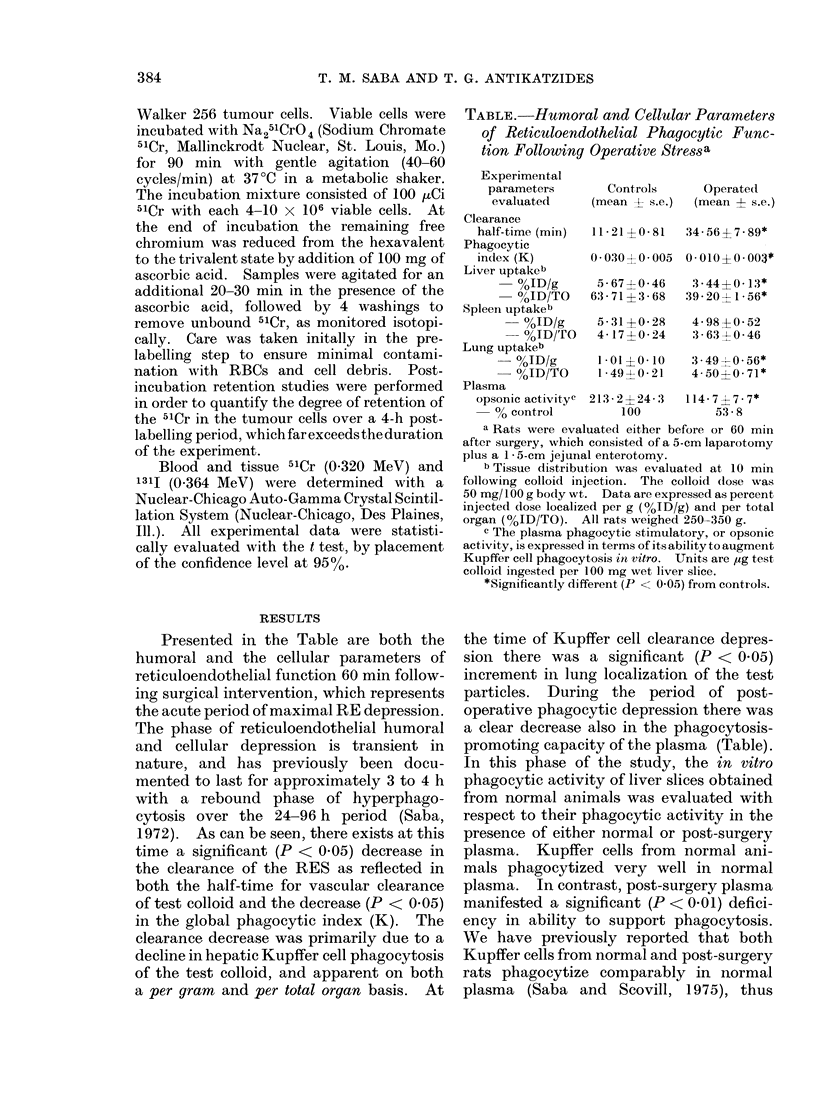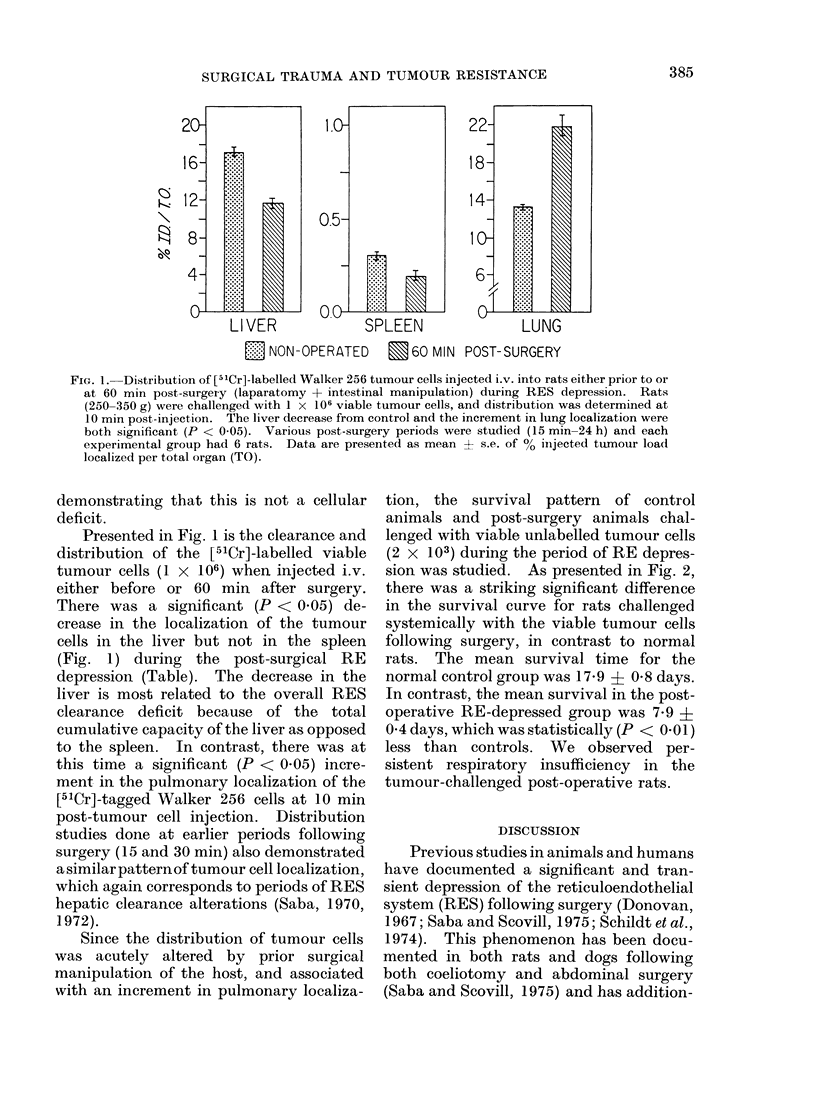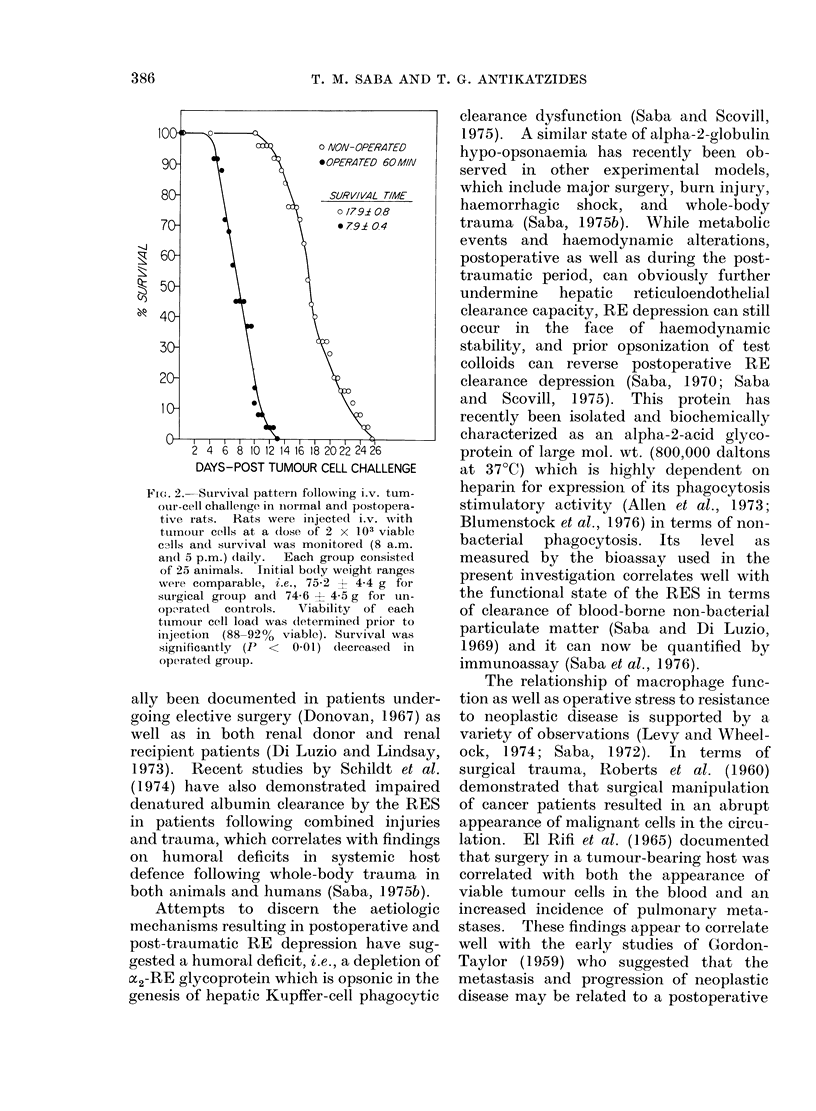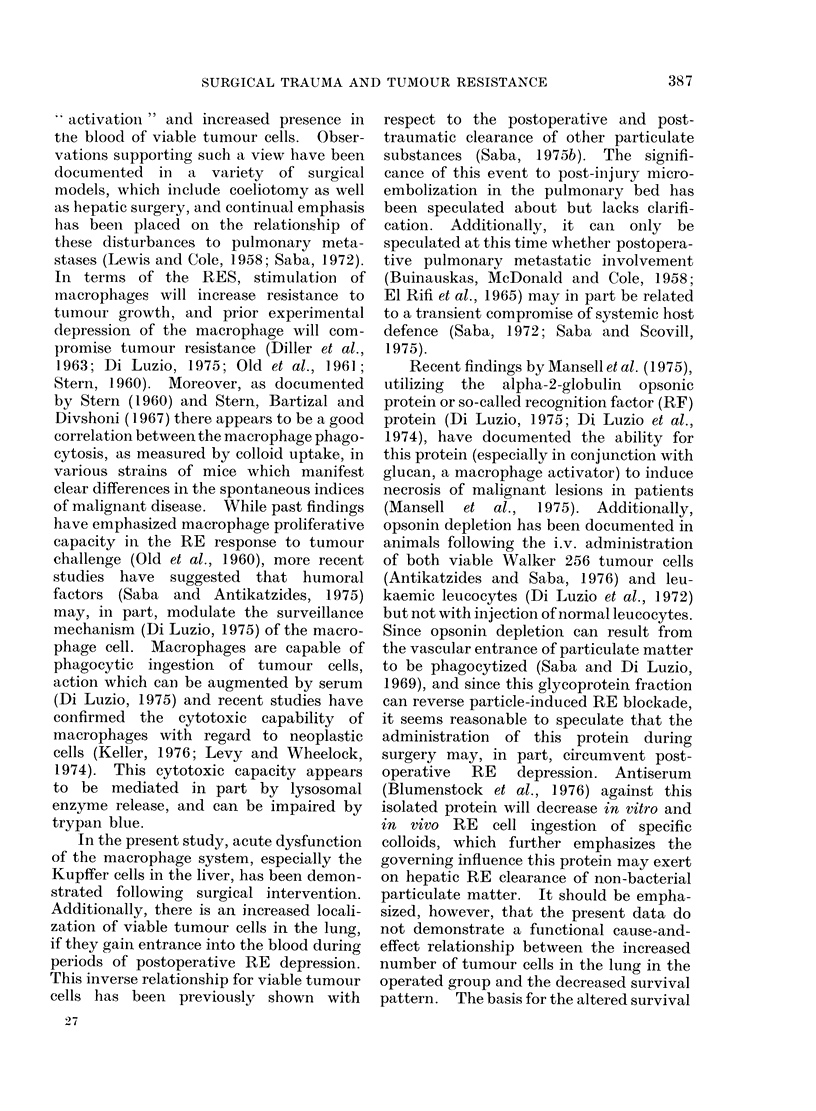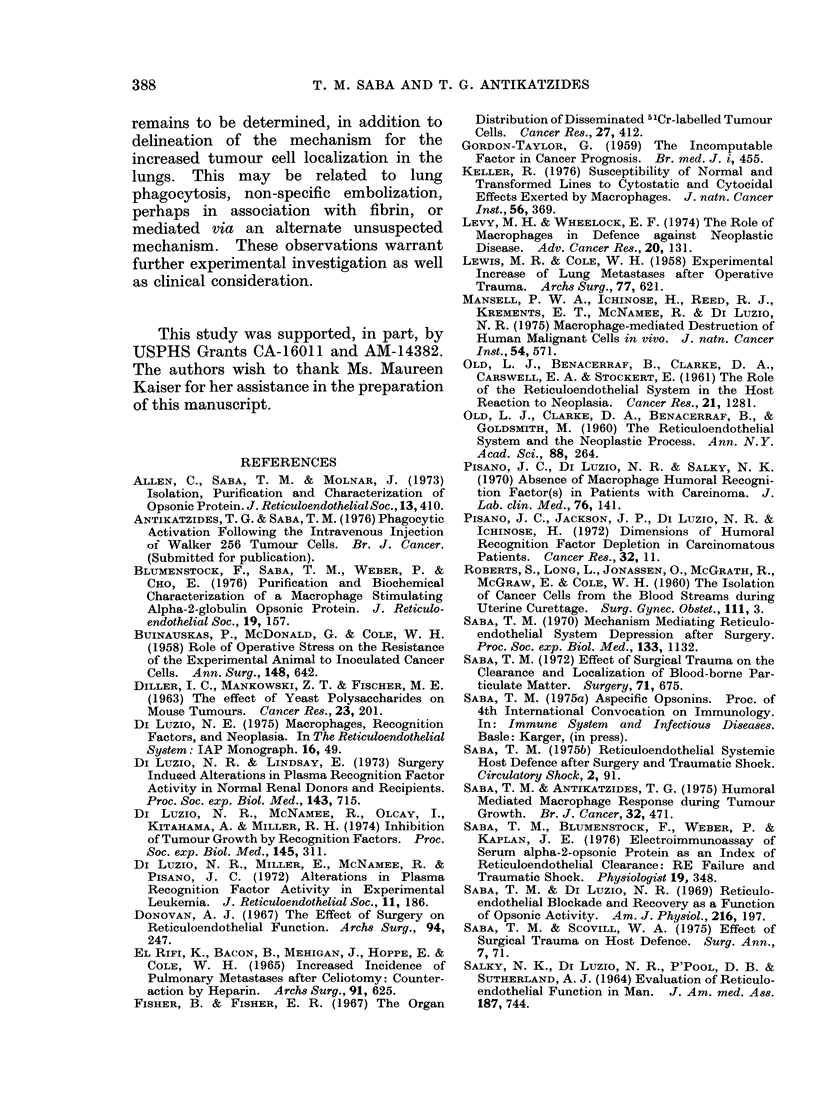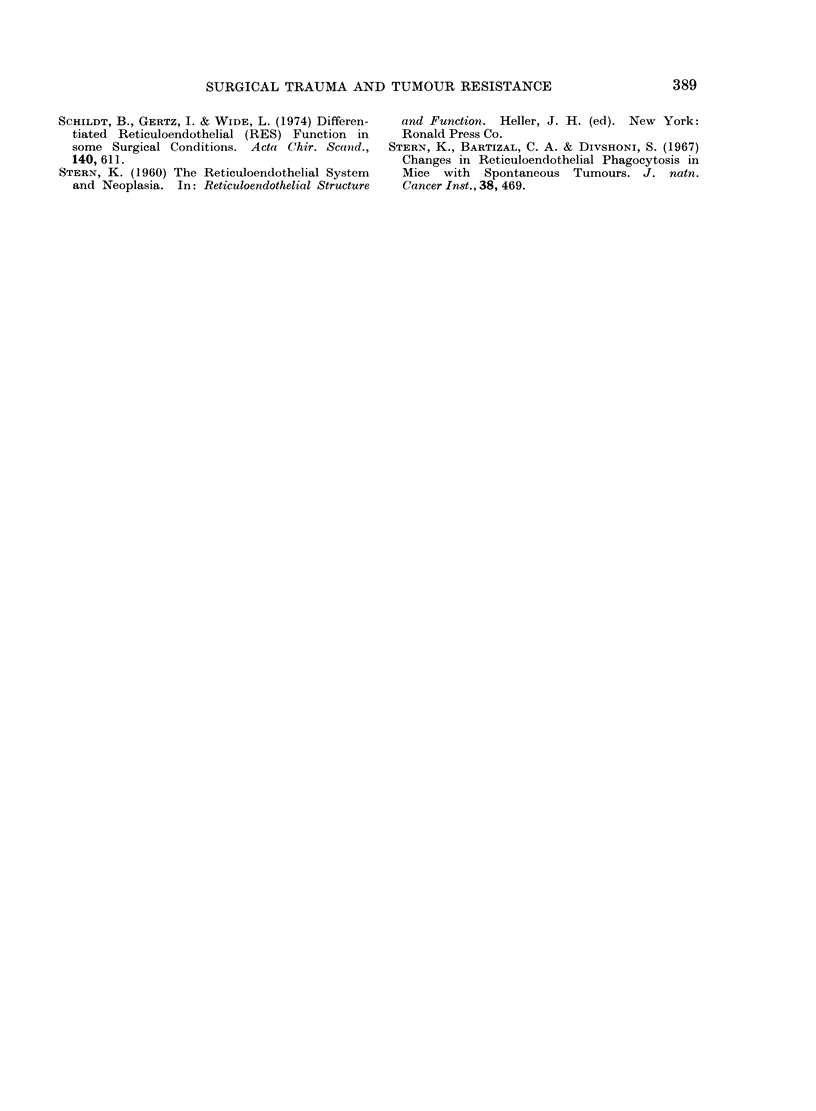# Decreased resistance to intravenous tumour-cell challenge during reticuloendothelial depression following surgery.

**DOI:** 10.1038/bjc.1976.181

**Published:** 1976-10

**Authors:** T. M. Saba, T. G. Antikatzides

## Abstract

The influence of surgical stress on resistance to i.v. challenge with Walker 256 tumour cells was investigated in rats, with respect to the functional state of the reticuloendothelial system (RES). Phagocytic activity of the RES was evaluated by colloid (gelatinized [131I] "RE test lipid emulsion") clearance, and opsonin levels were determined by bioassay. Reticuloendothelial clearance capacity was significantly (P less than 0-05) depressed 60 min following surgery (coeliotomy plus jejunal enterotomy) as quantified by both humoral and cellular parameters of RE function. Phagocytic depression was primarily due to impaired hepatic Kupffer cell function and related to a deficiency in the phagocytic supporting capacity of plasma, also referred to as opsonic or recognition factor (RF) capacity. During the postoperative period of RES colloid clearance depression, pulmonary localization of the blood-borne test particulate matter increased. Rats challenged with 51Cr-labelled viable tumour cells at a dose of 1-0 X 106 i.v., either prior to or during the postoperative period of RE depression, manifested a significant (P less than 0-05) increment in pulmonary localization of the viable tumour cells, and a decrease (P less than 0-05) in hepatic clearance. Evaluation of survival patterns demonstrated a significant (P less than 0-01) decrease in host resistance to i.v. tumour cell challenge (2 X 103 cells) during the postoperative period of RE depression and hypo-opsonaemia. Sham-anaesthetized control animals survived 17-9 +/- 0-8 days, while animals challenged during the period of RE depression survived 7-9 +/- 0-4 days. An increased incidence of respiratory distress and nasal discharge was observed in the animals with impaired survival. Thus, surgical manipulation may transiently compromise RES systemic host defence and may be reflected in an increment in the pulmonary localization of blood-borne tumour cells. The relationship of this altered pattern of tumour cell distribution to the impaired survival remains to be determined, and warrants investigations.


*Br. J. Cancer* (1976) **34,** 381

### DECREASED RESISTANCE TO INTRAVENOUS TUMOUR-CELL CHALLENGE DURING RETICULOENDOTHELIAL DEPRESSION FOLLOWING SURGERY[1]

T. M. SABA AND T. G. ANTIKATZIDES

*From the Department of Physiology, Albany Medical College of Union University, Albany, New York* 12208

Received 29 April 1976 Accepted 3 June 1976

**Summary.—The influence of surgical stress on resistance to i.v. challenge with Walker 256 tumour cells was investigated in rats, with respect to the functional state of the reticuloendothelial system (RES). Phagocytic activity of the RES was evaluated by colloid (gelatinized [$^{131}$I] " RE test lipid emulsion ") clearance, and opsonin levels were determined by bioassay. Reticuloendothelial clearance capacity was significantly ($P < 0.05$) depressed 60 min following surgery (coeliotomy plus jejunal enterotomy) as quantified by both humoral and cellular parameters of RE function. Phagocytic depression was primarily due to impaired hepatic Kupffer cell function and related to a deficiency in the phagocytic supporting capacity of plasma, also referred to as opsonic or recognition factor (RF) capacity. During the postoperative period of RES colloid clearance depression, pulmonary localization of the blood-borne test particulate matter increased. Rats challenged with $^{51}$Cr-labelled viable tumour cells at a dose of $1{\cdot}0 \times 10^6$ i.v., either prior to or during the postoperative period of RE depression, manifested a significant ($P < 0.05$) increment in pulmonary localization of the viable tumour cells, and a decrease ($P < 0.05$) in hepatic clearance. Evaluation of survival patterns demonstrated a significant ($P < 0.01$) decrease in host resistance to i.v. tumour cell challenge ($2 \times 10^3$ cells) during the postoperative period of RE depression and hypo-opsonaemia. Sham-anaesthetized control animals survived $17{\cdot}9 \pm 0{\cdot}8$ days, while animals challenged during the period of RE depression survived $7{\cdot}9 \pm 0{\cdot}4$ days. An increased incidence of respiratory distress and nasal discharge was observed in the animals with impaired survival. Thus, surgical manipulation may transiently compromise RES systemic host defence and may be reflected in an increment in the pulmonary localization of blood-borne tumour cells. The relationship of this altered pattern of tumour cell distribution to the impaired survival remains to be determined, and warrants investigation.**

THE IMPORTANCE of the reticuloendothelial system (RES) in host defence against bacterial infection, neoplastic disease, and traumatic shock has been well-documented (Di Luzio, 1975; Levy and Wheelock, 1974; Saba, 1975*b*). Such observations in both animals and humans emphasized the importance of systemic defence and the need to comprehend the factors that can alter its activity (Saba and Scovill, 1975; Schildt, Gertz and Wide, 1974). This view is supported by the experimental findings that RE stimulation can increase resistance to infection and tumour challenge and, furthermore, that experimental depression of the RES will compromise host survival to such insults (Diller, Mankowski and Fischer, 1963; Old *et al.*, 1960; Stern, 1960).

Previous studies from this laboratory (Saba, 1975*a*; Saba and Antikatzides, 1975) and others (Di Luzio, 1975; Levy and Wheelock, 1974) documented an important role for the macrophage system

Reprint requests to: Thomas M. Saba, Ph.D., Professor and Chairman, Department of Physiology, Albany Medical College of Union University, 47 New Scotland Avenue, Albany, New York 12208.

in the host response to neoplastic cells. Attempts to identify the factors modulating the phagocytic activity of the macrophage system, following tumour challenge as well as during metastatic disease, has previously emphasized the importance of the RE cell hypertrophy and hyperplasia (Old *et al.*, 1960, 1961). In contrast, recent data have emphasized the importance of a humoral factor in both the activation of macrophage activity and the development of terminal RES dysfunction (Di Luzio *et al.*, 1972, 1974; Saba and Antikatzides, 1975; Antikatzides and Saba, 1976). This humoral deficit during the later periods of tumour growth has been demonstrated in both animals (Saba and Antikatzides, 1975) and man (Pisano, Di Luzio and Salky, 1970; Pisano *et al.*, 1972) and is related to the blood level of a specific alpha-2-globulin, also called recognition factor or $\alpha_2$-RE glycoprotein, which has both opsonic and chemotactic properties (Allen, Saba and Molnar, 1973; Blumenstock *et al.*, 1976). This protein exerts a modulating influence on macrophage uptake of non-bacterial matter, both *in vitro* and *in vivo* (Blumenstock *et al.*, 1976; Di Luzio, 1975; Saba, 1975*a*), and disturbances of the RES systemic clearance capacity, following major surgery as well as traumatic shock, appears to be, in part, mediated by an acute but transient depletion and/or inhibition of this specific glycoprotein (Saba, 1970, 1972, 1975*b*) which can now be measured by immunoassay (Saba *et al.*, 1976).

Demonstration of a postoperative RE depression in conjunction with a deficiency of this opsonic alpha-2-glycoprotein has employed colloid clearance as an index of RE function (Saba, 1970, 1972; Saba and Scovill, 1975). The major organ involved in the clearance depression is the liver, and an inverse relationship appears to exist between hepatic clearance following both surgical trauma and whole body trauma and the level of pulmonary uptake of blood-borne particulate matter (Saba, 1975*b*). These observations, coupled with the finding that the entrance of neoplastic cells into the vascular compartment can deplete the plasma opsonin level (Antikatzides and Saba, 1976; Di Luzio *et al.*, 1972), suggest that postoperative hypo-opsonaemia and reticuloendothelial failure may precariously alter the systemic defence with respect to clearance and/or destruction of blood-borne tumour cells, and may be reflected in an increment in the pulmonary localization of viable tumour cells.

In the present study, experiments were designed to evaluate the concept that i.v. tumour cell challenge following surgery (during a period of postoperative RES depression) would be reflected in decreased liver uptake of these cells and an increment in pulmonary localization. The possibility that this event would reflect itself in an alteration in the survival rate to tumour cell challenge was also examined.

### EXPERIMENTAL METHODS

Male Holtzman rats maintained on Rockland Lab-Tek chow and tap water *ad libitum* were used in all studies. For *in vitro* liver slice phagocytic studies, normal rats employed as hepatic tissue donors were anaesthetized lightly with ether and rapidly desanguinated prior to liver removal. The excised liver was rapidly chilled in cold isotonic saline, prior to liver slice preparation with the use of a Stadie-Riggs tissue slicer, as previously described (Allen *et al.*, 1973; Blumenstock *et al.*, 1976; Pisano *et al.*, 1970; Saba and Di Luzio, 1969). Plasma bioassayed for phagocytic stimulatory activity was obtained by vena cava puncture and maintained briefly at 4°C prior to evaluation.

The surgical stress consisted of a mid-line laparotomy approximately 5 cm long, coupled with gentle intestinal manipulation for 15–30 s or a 1·5-cm jejunal incision under light ether anaesthesia (Saba, 1972). Following the surgical procedure, the incision was rapidly closed with 6–0 silk suture, utilizing an interrupted stitch, and the anaesthesia was terminated. Control rats were exposed to similar conditions of anaesthetization. Following surgery, the rats were mobile within a few minutes after termination of the anaesthesia.

Reticuloendothelial (RE) phagocytic function was assessed with a colloid clearance technique coupled with *in vivo* colloid distribution studies (Saba and Antikatzides, 1975; Saba and Di Luzio, 1969; Salky *et al.*, 1964). The [$^{131}$I] gelatinized " RE test lipid emulsion ", previously shown to be selectively removed by the process of phagocytosis, especially by the liver and spleen, was utilized (Saba and Di Luzio, 1969; Salky *et al.*, 1964). The emulsion was prepared from glycerol, [$^{131}$I] triolein (Mallinckrodt Nuclear, St. Louis, Mo.) and lecithin, mixed in a ratio of 10 : 10 : 1 by weight, respectively. Prior to i.v. injection, the emulsion base was incubated at 37°C for 20 min in a 0·3% gelatin-supplemented sterile 5% dextrose and water solution previously adjusted to a pH of 7·4. The lipid emulsion used *in vivo* for measurement of RE clearance function had a base concentration of 10% and was injected i.v. at a dose of 50 mg/100 g body wt. Phagocytic activity was measured by the half-time (t/2) for the vascular clearance of the test colloid, and [$^{131}$I] colloid levels in the blood were assayed on 5 serial 0·1-ml samples of whole blood obtained by tail vein puncture. Blood [$^{131}$I] radioactivity was plotted semi-logarithmically against time in minutes, and clearance half-times (t/2) as well as the global phagocytic index (K) for the vascular phagocytic removal of the colloid were determined. At 10 min post-injection, distribution of the test particle in the liver, lungs, and spleen was evaluated (Saba and Di Luzio, 1969) on a wet weight basis, and expressed as the percentage of the injected dose phagocytized per gram (%ID/g) and per total organ (%ID/TO).

Phagocytic stimulatory or opsonic capacity of plasma was determined with a liver slice technique, in which plasma from normal rats and from rats at 60 min post-surgery during RE depression was comparatively evaluated for its ability to stimulate hepatic Kupffer cell phagocytosis (Blumenstock *et al.*, 1976; Mansell *et al.*, 1975; Saba and Di Luzio, 1969; Saba and Antikatzides, 1975). The incubation system consisted of 3 ml of heparinized (50 USP u/ml) control or experimental plasma medium, 2000 $\mu$g of the [$^{131}$I] lipid emulsion (1% lipid emulsion with 0·1% gelatin), and liver slices, as previously described (Saba and Di Luzio, 1969). All incubation samples were gassed with 95% $O_2$ and 5% $CO_2$ and incubated at 37°C for 30 min. At the end of the incubation, the liver slices were removed, briefly washed in cold isotonic saline, and analysed for Kupffer cell colloid uptake. Phagocytosis was expressed as both the percentage of the injected dose and $\mu$g of colloid phagocytized per 100 mg of tissue. This technique has been previously used to assay the level of this phagocytic stimulatory protein (alpha-2-globulin opsonic protein) in animals and man (Di Luzio, 1975; Pisano *et al.*, 1970; Saba, 1970, 1972) and the precision of the assay for quantifying blood level alterations has recently been confirmed by immunoassay, utilizing monospecific antiserum to the isolated opsonic alpha-2-glycoprotein (Blumenstock *et al.*, 1976).

For tumour colony maintenance, male Holtzman rats weighing 60–70 g and approximately 22–30 days of age were used in all experiments as recipients (Saba and Antikatzides, 1975). They were maintained on Tek-Lab chow and tap water *ad libitum* before and following tumour transplantation. Walker 256 donor tumour-bearing rats were originally obtained from Microbiological Associates, Inc. (Bethesda, Maryland) and the tumour was subsequently maintained by serial transplantation at 10–12-day intervals. The serial transplantation of the Walker 256 tumour was accomplished as previously described (Antikatzides and Saba, 1976; Saba and Antikatzides, 1975) using a tumour load of $2 \times 10^4$ viable cells intramuscularly. Tumour donors were anaesthetized by light ether anaesthesia and the tumour was excised in a sterile transplantation box. The viable periphery of the tumour mass was passed through a No. 8, 177-$\mu$m pore microsieve and cells were collected in sterile saline and analyzed for viability by dye exclusion. Utilizing this procedure, there is a 98% " take " rate in terms of tumour growth (Saba and Antikatzides, 1975) with a relatively uniform growth rate.

For the surgical studies in terms of survival to tumour-cell challenge, each pre- and post-operative experimental recipient rat received $2 \times 10^3$ viable cells i.v. in a volume of 0·2 ml of sterile saline. Controls were anaesthetized and injected with 0·2 ml saline. For distribution studies with the [$^{51}$Cr]-labelled tumour cells, a load of $1 \cdot 0 \times 10^6$ viable cells (i.v.) was used in a volume of 0·5 ml saline, and controls received 0·5 ml saline. The technique for $^{51}$[Cr]-labelling of the tumour cells was very similar to that used by Fisher and Fisher (1967) for the labelling of

Walker 256 tumour cells. Viable cells were incubated with $Na_2{}^{51}CrO_4$ (Sodium Chromate $^{51}Cr$, Mallinckrodt Nuclear, St. Louis, Mo.) for 90 min with gentle agitation (40–60 cycles/min) at 37 °C in a metabolic shaker. The incubation mixture consisted of 100 $\mu$Ci $^{51}Cr$ with each 4–10 $\times$ $10^6$ viable cells. At the end of incubation the remaining free chromium was reduced from the hexavalent to the trivalent state by addition of 100 mg of ascorbic acid. Samples were agitated for an additional 20–30 min in the presence of the ascorbic acid, followed by 4 washings to remove unbound $^{51}Cr$, as monitored isotopically. Care was taken initally in the prelabelling step to ensure minimal contamination with RBCs and cell debris. Post-incubation retention studies were performed in order to quantify the degree of retention of the $^{51}Cr$ in the tumour cells over a 4-h post-labelling period, which far exceeds the duration of the experiment.

Blood and tissue $^{51}Cr$ (0·320 MeV) and $^{131}I$ (0·364 MeV) were determined with a Nuclear-Chicago Auto-Gamma Crystal Scintillation System (Nuclear-Chicago, Des Plaines, Ill.). All experimental data were statistically evaluated with the *t* test, by placement of the confidence level at 95%.

TABLE.—*Humoral and Cellular Parameters of Reticuloendothelial Phagocytic Function Following Operative Stress*[a]

| Experimental parameters evaluated | Controls (mean ± s.e.) | Operated (mean ± s.e.) |
|---|---|---|
| Clearance half-time (min) | 11·21±0·81 | 34·56±7·89* |
| Phagocytic index (K) | 0·030±0·005 | 0·010±0·003* |
| Liver uptake[b] | | |
| — %ID/g | 5·67±0·46 | 3·44±0·13* |
| — %ID/TO | 63·71±3·68 | 39·20±1·56* |
| Spleen uptake[b] | | |
| — %ID/g | 5·31±0·28 | 4·98±0·52 |
| — %ID/TO | 4·17±0·24 | 3·63±0·46 |
| Lung uptake[b] | | |
| — %ID/g | 1·01±0·10 | 3·49±0·56* |
| — %ID/TO | 1·49±0·21 | 4·50±0·71* |
| Plasma opsonic activity[c] | 213·2±24·3 | 114·7±7·7* |
| — % control | 100 | 53·8 |

[a] Rats were evaluated either before or 60 min after surgery, which consisted of a 5-cm laparotomy plus a 1·5-cm jejunal enterotomy.

[b] Tissue distribution was evaluated at 10 min following colloid injection. The colloid dose was 50 mg/100 g body wt. Data are expressed as percent injected dose localized per g (%ID/g) and per total organ (%ID/TO). All rats weighed 250–350 g.

[c] The plasma phagocytic stimulatory, or opsonic activity, is expressed in terms of its ability to augment Kupffer cell phagocytosis *in vitro*. Units are $\mu$g test colloid ingested per 100 mg wet liver slice.

*Significantly different ($P < 0·05$) from controls.

### RESULTS

Presented in the Table are both the humoral and the cellular parameters of reticuloendothelial function 60 min following surgical intervention, which represents the acute period of maximal RE depression. The phase of reticuloendothelial humoral and cellular depression is transient in nature, and has previously been documented to last for approximately 3 to 4 h with a rebound phase of hyperphagocytosis over the 24–96 h period (Saba, 1972). As can be seen, there exists at this time a significant ($P < 0·05$) decrease in the clearance of the RES as reflected in both the half-time for vascular clearance of test colloid and the decrease ($P < 0·05$) in the global phagocytic index (K). The clearance decrease was primarily due to a decline in hepatic Kupffer cell phagocytosis of the test colloid, and apparent on both a *per gram* and *per total organ* basis. At the time of Kupffer cell clearance depression there was a significant ($P < 0·05$) increment in lung localization of the test particles. During the period of post-operative phagocytic depression there was a clear decrease also in the phagocytosis-promoting capacity of the plasma (Table). In this phase of the study, the *in vitro* phagocytic activity of liver slices obtained from normal animals was evaluated with respect to their phagocytic activity in the presence of either normal or post-surgery plasma. Kupffer cells from normal animals phagocytized very well in normal plasma. In contrast, post-surgery plasma manifested a significant ($P < 0·01$) deficiency in ability to support phagocytosis. We have previously reported that both Kupffer cells from normal and post-surgery rats phagocytize comparably in normal plasma (Saba and Scovill, 1975), thus

FIG. 1.—Distribution of [$^{51}$Cr]-labelled Walker 256 tumour cells injected i.v. into rats either prior to or at 60 min post-surgery (laparatomy + intestinal manipulation) during RES depression. Rats (250–350 g) were challenged with $1 \times 10^6$ viable tumour cells, and distribution was determined at 10 min post-injection. The liver decrease from control and the increment in lung localization were both significant ($P < 0{\cdot}05$). Various post-surgery periods were studied (15 min–24 h) and each experimental group had 6 rats. Data are presented as mean ± s.e. of % injected tumour load localized per total organ (TO).

demonstrating that this is not a cellular deficit.

Presented in Fig. 1 is the clearance and distribution of the [$^{51}$Cr]-labelled viable tumour cells ($1 \times 10^6$) when injected i.v. either before or 60 min after surgery. There was a significant ($P < 0{\cdot}05$) decrease in the localization of the tumour cells in the liver but not in the spleen (Fig. 1) during the post-surgical RE depression (Table). The decrease in the liver is most related to the overall RES clearance deficit because of the total cumulative capacity of the liver as opposed to the spleen. In contrast, there was at this time a significant ($P < 0{\cdot}05$) increment in the pulmonary localization of the [$^{51}$Cr]-tagged Walker 256 cells at 10 min post-tumour cell injection. Distribution studies done at earlier periods following surgery (15 and 30 min) also demonstrated a similar pattern of tumour cell localization, which again corresponds to periods of RES hepatic clearance alterations (Saba, 1970, 1972).

Since the distribution of tumour cells was acutely altered by prior surgical manipulation of the host, and associated with an increment in pulmonary localization, the survival pattern of control animals and post-surgery animals challenged with viable unlabelled tumour cells ($2 \times 10^3$) during the period of RE depression was studied. As presented in Fig. 2, there was a striking significant difference in the survival curve for rats challenged systemically with the viable tumour cells following surgery, in contrast to normal rats. The mean survival time for the normal control group was 17·9 ± 0·8 days. In contrast, the mean survival in the post-operative RE-depressed group was 7·9 ± 0·4 days, which was statistically ($P < 0{\cdot}01$) less than controls. We observed persistent respiratory insufficiency in the tumour-challenged post-operative rats.

DISCUSSION

Previous studies in animals and humans have documented a significant and transient depression of the reticuloendothelial system (RES) following surgery (Donovan, 1967; Saba and Scovill, 1975; Schildt *et al.*, 1974). This phenomenon has been documented in both rats and dogs following both coeliotomy and abdominal surgery (Saba and Scovill, 1975) and has addition-

FIG. 2.—Survival pattern following i.v. tumour-cell challenge in normal and postoperative rats. Rats were injected i.v. with tumour cells at a dose of $2 \times 10^3$ viable cells and survival was monitored (8 a.m. and 5 p.m.) daily. Each group consisted of 25 animals. Initial body weight ranges were comparable, *i.e.*, 75·2 ± 4·4 g for surgical group and 74·6 ± 4·5 g for unoperated controls. Viability of each tumour cell load was determined prior to injection (88–92% viable). Survival was significantly ($P < 0·01$) decreased in operated group.

ally been documented in patients undergoing elective surgery (Donovan, 1967) as well as in both renal donor and renal recipient patients (Di Luzio and Lindsay, 1973). Recent studies by Schildt *et al.* (1974) have also demonstrated impaired denatured albumin clearance by the RES in patients following combined injuries and trauma, which correlates with findings on humoral deficits in systemic host defence following whole-body trauma in both animals and humans (Saba, 1975*b*).

Attempts to discern the aetiologic mechanisms resulting in postoperative and post-traumatic RE depression have suggested a humoral deficit, *i.e.*, a depletion of $\alpha_2$-RE glycoprotein which is opsonic in the genesis of hepatic Kupffer-cell phagocytic clearance dysfunction (Saba and Scovill, 1975). A similar state of alpha-2-globulin hypo-opsonaemia has recently been observed in other experimental models, which include major surgery, burn injury, haemorrhagic shock, and whole-body trauma (Saba, 1975*b*). While metabolic events and haemodynamic alterations, postoperative as well as during the post-traumatic period, can obviously further undermine hepatic reticuloendothelial clearance capacity, RE depression can still occur in the face of haemodynamic stability, and prior opsonization of test colloids can reverse postoperative RE clearance depression (Saba, 1970; Saba and Scovill, 1975). This protein has recently been isolated and biochemically characterized as an alpha-2-acid glycoprotein of large mol. wt. (800,000 daltons at 37°C) which is highly dependent on heparin for expression of its phagocytosis stimulatory activity (Allen *et al.*, 1973; Blumenstock *et al.*, 1976) in terms of non-bacterial phagocytosis. Its level as measured by the bioassay used in the present investigation correlates well with the functional state of the RES in terms of clearance of blood-borne non-bacterial particulate matter (Saba and Di Luzio, 1969) and it can now be quantified by immunoassay (Saba *et al.*, 1976).

The relationship of macrophage function as well as operative stress to resistance to neoplastic disease is supported by a variety of observations (Levy and Wheelock, 1974; Saba, 1972). In terms of surgical trauma, Roberts *et al.* (1960) demonstrated that surgical manipulation of cancer patients resulted in an abrupt appearance of malignant cells in the circulation. El Rifi *et al.* (1965) documented that surgery in a tumour-bearing host was correlated with both the appearance of viable tumour cells in the blood and an increased incidence of pulmonary metastases. These findings appear to correlate well with the early studies of Gordon-Taylor (1959) who suggested that the metastasis and progression of neoplastic disease may be related to a postoperative

"activation" and increased presence in the blood of viable tumour cells. Observations supporting such a view have been documented in a variety of surgical models, which include coeliotomy as well as hepatic surgery, and continual emphasis has been placed on the relationship of these disturbances to pulmonary metastases (Lewis and Cole, 1958; Saba, 1972). In terms of the RES, stimulation of macrophages will increase resistance to tumour growth, and prior experimental depression of the macrophage will compromise tumour resistance (Diller *et al.*, 1963; Di Luzio, 1975; Old *et al.*, 1961; Stern, 1960). Moreover, as documented by Stern (1960) and Stern, Bartizal and Divshoni (1967) there appears to be a good correlation between the macrophage phagocytosis, as measured by colloid uptake, in various strains of mice which manifest clear differences in the spontaneous indices of malignant disease. While past findings have emphasized macrophage proliferative capacity in the RE response to tumour challenge (Old *et al.*, 1960), more recent studies have suggested that humoral factors (Saba and Antikatzides, 1975) may, in part, modulate the surveillance mechanism (Di Luzio, 1975) of the macrophage cell. Macrophages are capable of phagocytic ingestion of tumour cells, action which can be augmented by serum (Di Luzio, 1975) and recent studies have confirmed the cytotoxic capability of macrophages with regard to neoplastic cells (Keller, 1976; Levy and Wheelock, 1974). This cytotoxic capacity appears to be mediated in part by lysosomal enzyme release, and can be impaired by trypan blue.

In the present study, acute dysfunction of the macrophage system, especially the Kupffer cells in the liver, has been demonstrated following surgical intervention. Additionally, there is an increased localization of viable tumour cells in the lung, if they gain entrance into the blood during periods of postoperative RE depression. This inverse relationship for viable tumour cells has been previously shown with respect to the postoperative and post-traumatic clearance of other particulate substances (Saba, 1975*b*). The significance of this event to post-injury microembolization in the pulmonary bed has been speculated about but lacks clarification. Additionally, it can only be speculated at this time whether postoperative pulmonary metastatic involvement (Buinauskas, McDonald and Cole, 1958; El Rifi *et al.*, 1965) may in part be related to a transient compromise of systemic host defence (Saba, 1972; Saba and Scovill, 1975).

Recent findings by Mansell *et al.* (1975), utilizing the alpha-2-globulin opsonic protein or so-called recognition factor (RF) protein (Di Luzio, 1975; Di Luzio *et al.*, 1974), have documented the ability for this protein (especially in conjunction with glucan, a macrophage activator) to induce necrosis of malignant lesions in patients (Mansell *et al.*, 1975). Additionally, opsonin depletion has been documented in animals following the i.v. administration of both viable Walker 256 tumour cells (Antikatzides and Saba, 1976) and leukaemic leucocytes (Di Luzio *et al.*, 1972) but not with injection of normal leucocytes. Since opsonin depletion can result from the vascular entrance of particulate matter to be phagocytized (Saba and Di Luzio, 1969), and since this glycoprotein fraction can reverse particle-induced RE blockade, it seems reasonable to speculate that the administration of this protein during surgery may, in part, circumvent postoperative RE depression. Antiserum (Blumenstock *et al.*, 1976) against this isolated protein will decrease *in vitro* and *in vivo* RE cell ingestion of specific colloids, which further emphasizes the governing influence this protein may exert on hepatic RE clearance of non-bacterial particulate matter. It should be emphasized, however, that the present data do not demonstrate a functional cause-and-effect relationship between the increased number of tumour cells in the lung in the operated group and the decreased survival pattern. The basis for the altered survival remains to be determined, in addition to delineation of the mechanism for the increased tumour cell localization in the lungs. This may be related to lung phagocytosis, non-specific embolization, perhaps in association with fibrin, or mediated *via* an alternate unsuspected mechanism. These observations warrant further experimental investigation as well as clinical consideration.

This study was supported, in part, by USPHS Grants CA-16011 and AM-14382. The authors wish to thank Ms. Maureen Kaiser for her assistance in the preparation of this manuscript.

### REFERENCES

ALLEN, C., SABA, T. M. & MOLNAR, J. (1973) Isolation, Purification and Characterization of Opsonic Protein. *J. Reticuloendothelial Soc.*, **13**, 410.

ANTIKATZIDES, T. G. & SABA, T. M. (1976) Phagocytic Activation Following the Intravenous Injection of Walker 256 Tumour Cells. *Br. J. Cancer.* (Submitted for publication).

BLUMENSTOCK, F., SABA, T. M., WEBER, P. & CHO, E. (1976) Purification and Biochemical Characterization of a Macrophage Stimulating Alpha-2-globulin Opsonic Protein. *J. Reticuloendothelial Soc.*, **19**, 157.

BUINAUSKAS, P., McDONALD, G. & COLE, W. H. (1958) Role of Operative Stress on the Resistance of the Experimental Animal to Inoculated Cancer Cells. *Ann. Surg.*, **148**, 642.

DILLER, I. C., MANKOWSKI, Z. T. & FISCHER, M. E. (1963) The effect of Yeast Polysaccharides on Mouse Tumours. *Cancer Res.*, **23**, 201.

DI LUZIO, N. E. (1975) Macrophages, Recognition Factors, and Neoplasia. In *The Reticuloendothelial System*: IAP Monograph. **16**, 49.

DI LUZIO, N. R. & LINDSAY, E. (1973) Surgery Induced Alterations in Plasma Recognition Factor Activity in Normal Renal Donors and Recipients. *Proc. Soc. exp. Biol. Med.*, **143**, 715.

DI LUZIO, N. R., McNAMEE, R., OLCAY, I., KITAHAMA, A. & MILLER, R. H. (1974) Inhibition of Tumour Growth by Recognition Factors. *Proc. Soc. exp. Biol. Med.*, **145**, 311.

DI LUZIO, N. R., MILLER, E., McNAMEE, R. & PISANO, J. C. (1972) Alterations in Plasma Recognition Factor Activity in Experimental Leukemia. *J. Reticuloendothelial Soc.*, **11**, 186.

DONOVAN, A. J. (1967) The Effect of Surgery on Reticuloendothelial Function. *Archs Surg.*, **94**, 247.

EL RIFI, K., BACON, B., MEHIGAN, J., HOPPE, E. & COLE, W. H. (1965) Increased Incidence of Pulmonary Metastases after Celiotomy: Counteraction by Heparin. *Archs Surg.*, **91**, 625.

FISHER, B. & FISHER, E. R. (1967) The Organ Distribution of Disseminated $^{51}$Cr-labelled Tumour Cells. *Cancer Res.*, **27**, 412.

GORDON-TAYLOR, G. (1959) The Incomputable Factor in Cancer Prognosis. *Br. med. J. i*, 455.

KELLER, R. (1976) Susceptibility of Normal and Transformed Lines to Cytostatic and Cytocidal Effects Exerted by Macrophages. *J. natn. Cancer Inst.*, **56**, 369.

LEVY, M. H. & WHEELOCK, E. F. (1974) The Role of Macrophages in Defence against Neoplastic Disease. *Adv. Cancer Res.*, **20**, 131.

LEWIS, M. R. & COLE, W. H. (1958) Experimental Increase of Lung Metastases after Operative Trauma. *Archs Surg.*, **77**, 621.

MANSELL, P. W. A., ICHINOSE, H., REED, R. J., KREMENTS, E. T., McNAMEE, R. & DI LUZIO, N. R. (1975) Macrophage-mediated Destruction of Human Malignant Cells *in vivo*. *J. natn. Cancer Inst.*, **54**, 571.

OLD, L. J., BENACERRAF, B., CLARKE, D. A., CARSWELL, E. A. & STOCKERT, E. (1961) The Role of the Reticuloendothelial System in the Host Reaction to Neoplasia. *Cancer Res.*, **21**, 1281.

OLD, L. J., CLARKE, D. A., BENACERRAF, B., & GOLDSMITH, M. (1960) The Reticuloendothelial System and the Neoplastic Process. *Ann. N.Y. Acad. Sci.*, **88**, 264.

PISANO, J. C., DI LUZIO, N. R. & SALKY, N. K. (1970) Absence of Macrophage Humoral Recognition Factor(s) in Patients with Carcinoma. *J. Lab. clin. Med.*, **76**, 141.

PISANO, J. C., JACKSON, J. P., DI LUZIO, N. R. & ICHINOSE, H. (1972) Dimensions of Humoral Recognition Factor Depletion in Carcinomatous Patients. *Cancer Res.*, **32**, 11.

ROBERTS, S., LONG, L., JONASSEN, O., McGRATH, R., McGRAW, E. & COLE, W. H. (1960) The Isolation of Cancer Cells from the Blood Streams during Uterine Curettage. *Surg. Gynec. Obstet.*, **111**, 3.

SABA, T. M. (1970) Mechanism Mediating Reticuloendothelial System Depression after Surgery. *Proc. Soc. exp. Biol. Med.*, **133**, 1132.

SABA, T. M. (1972) Effect of Surgical Trauma on the Clearance and Localization of Blood-borne Particulate Matter. *Surgery*, **71**, 675.

SABA, T. M. (1975*a*) Aspecific Opsonins. Proc. of 4th International Convocation on Immunology. In: *Immune System and Infectious Diseases.* Basle: Karger, (in press).

SABA, T. M. (1975*b*) Reticuloendothelial Systemic Host Defence after Surgery and Traumatic Shock. *Circulatory Shock*, **2**, 91.

SABA, T. M. & ANTIKATZIDES, T. G. (1975) Humoral Mediated Macrophage Response during Tumour Growth. *Br. J. Cancer*, **32**, 471.

SABA, T. M., BLUMENSTOCK, F., WEBER, P. & KAPLAN, J. E. (1976) Electroimmunoassay of Serum alpha-2-opsonic Protein as an Index of Reticuloendothelial Clearance: RE Failure and Traumatic Shock. *Physiologist* **19**, 348.

SABA, T. M. & DI LUZIO, N. R. (1969) Reticuloendothelial Blockade and Recovery as a Function of Opsonic Activity. *Am. J. Physiol.*, **216**, 197.

SABA, T. M. & SCOVILL, W. A. (1975) Effect of Surgical Trauma on Host Defence. *Surg. Ann.*, **7**, 71.

SALKY, N. K., DI LUZIO, N. R., P'POOL, D. B. & SUTHERLAND, A. J. (1964) Evaluation of Reticuloendothelial Function in Man. *J. Am. med. Ass.* **187**, 744.

SCHILDT, B., GERTZ, I. & WIDE, L. (1974) Differentiated Reticuloendothelial (RES) Function in some Surgical Conditions. *Acta Chir. Scand.*, **140,** 611.

STERN, K. (1960) The Reticuloendothelial System and Neoplasia. In: *Reticuloendothelial Structure and Function*. Heller, J. H. (ed). New York: Ronald Press Co.

STERN, K., BARTIZAL, C. A. & DIVSHONI, S. (1967) Changes in Reticuloendothelial Phagocytosis in Mice with Spontaneous Tumours. *J. natn. Cancer Inst.*, **38,** 469.